# GFF-Ex: a genome feature extraction package

**DOI:** 10.1186/1756-0500-7-315

**Published:** 2014-05-24

**Authors:** Achal Rastogi, Dinesh Gupta

**Affiliations:** 1 Bioinformatics Laboratory, Structural and Computational Biology Group, International Center for Genetic Engineering and Biotechnology, Aruna Asaf Ali Marg, New Delhi 110067, India

**Keywords:** GFF, Genomics, Annotation, Sequence parser

## Abstract

**Background:**

Genomic features of whole genome sequences emerging from various sequencing and annotation projects are represented and stored in several formats. Amongst these formats, the GFF (Generic/General Feature Format) has emerged as a widely accepted, portable and successfully used flat file format for genome annotation storage. With an increasing interest in genome annotation projects and secondary and meta-analysis, there is a need for efficient tools to extract sequences of interests from GFF files.

**Findings:**

We have developed GFF-Ex to automate feature-based extraction of sequences from a GFF file. In addition to automated sequence extraction of the features described within a feature file, GFF-Ex also assigns boundaries for the features (introns, intergenic, regions upstream to genes), which are not explicitly specified in the GFF format, and exports the corresponding primary sequence information into predefined feature specific output files. GFF-Ex package consists of several UNIX Shell and PERL scripts.

**Conclusions:**

Compared to other available GFF parsers, GFF-Ex is a simpler tool, which permits sequence retrieval based on additional inferred features. GFF-Ex can also be integrated with any genome annotation or analysis pipeline. GFF-Ex is freely available at http://bioinfo.icgeb.res.in/gff.

## Findings

**Figure 1:**
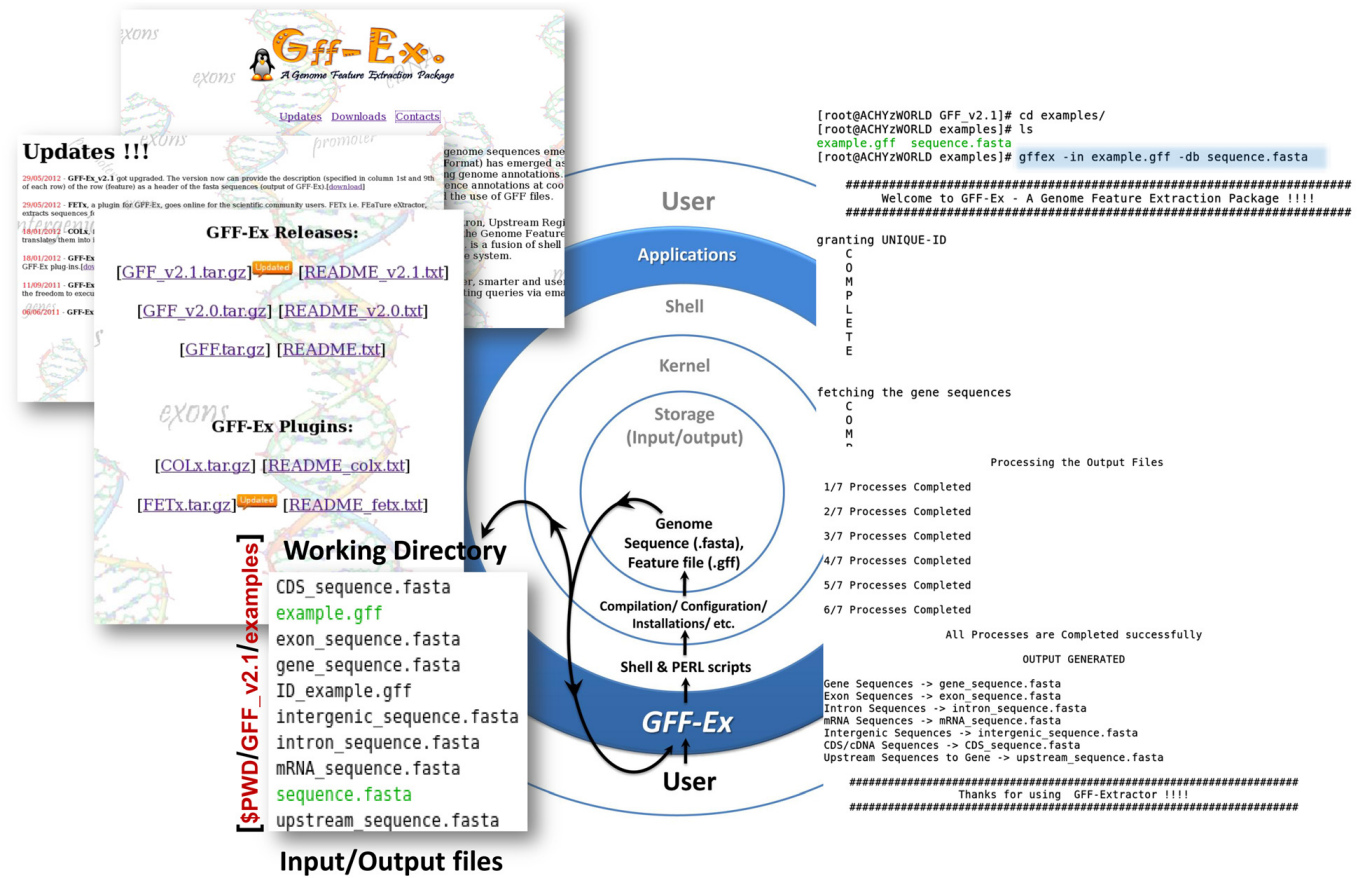
**GFF-Ex flow.** Usage of each component of the Operating System (UNIX) at various levels of applications execution. GFF-Ex is executed through Shell. GFF-Ex takes multiple inputs and arguments from the working directory, processes the files in kernel and produces the output back to the working directory, mounted over the storage. The flow is iterated till each component of GFF-Ex is executed, producing desirable results.

Technical advancements in the field of high-throughput DNA sequencing [1,2], the ease of performing experiments, rapid development of tools and convenient accessibility of various bioinformatics resources [3,4] are some of the major key factors that have resulted in worldwide increase in sequencing and annotation projects [5,6]. General Feature Format/Generic Feature Format (GFF) (http://www.sanger.ac.uk/resources/software/gff/spec.html) is a flat file data format widely used for storing genome annotations, describing sequence-based annotations of a genome. GFF represents genome feature data in a tab-delimited table, single feature per line, making it ideal for use with various data analysis pipelines. Currently, the scientific communities are becoming more and more reliant on these information for secondary analysis and laboratory experiments too, which has resulted in a growing need for efficient tools to extract desirable sequences based on annotations [7]. This motivated us to develop GFF-Ex. GFF-Ex is a genome feature based sequence extraction package to automate sequence extraction based on the features defined within feature files (Figure 1). GFF-Ex works on OS platforms with UNIX file systems. For GFF-Ex development, source code testing and analysis of package performance was conducted on a Sun Ultra 27 Workstation with 3GB of memory (RAM), powered with a Xeon processor running on 2.66 GHz. GFF-Ex source code consists of several Shell and PERL scripts. The PERL scripts within a Shell script framework maintain the flow of the entire algorithm. Structured package within Shell framework renders enhancement of GFF-Ex performance and speed, as it allows input–output and inter-process communication parsed through UNIX pipes. The pipes allow information to pass in memory between consecutive steps in a pipeline of programs being run, and not being written to disk for downstream processing. Thus, by using the pipeline described above, we were able to save system memory and time for I/O operations, enhancing the package performance.

### GFF-Ex

GFF-Ex (http://bioinfo.icgeb.res.in/gff/) is a collection of various modules developed using Shell and PERL string parsing scripts. The current version of GFF-Ex (version 2.2, http://bioinfo.icgeb.res.in/gff/gffdownloads/GFF_v2.2.tar.gz) equipped with an installation file, copies program scripts to the user specified installation directory. The package also includes few example files and a README file to help users install and execute GFF-Ex. GFF-Ex is robust and adaptable towards future integrations of the GFF-Ex plug-ins. The software enables GFF-Ex users to pass input arguments and parameters as a single command statement, along with invoking GFF-Ex. This helps GFF-Ex to switch to the specified modules/plug-ins without taking much of kernel time. For an input file size of about 500 MB, the CPU time between invocation and termination of GFF-Ex is 2 m2.305 s. The current GFF-Ex architecture is suitable for transparent integration with various sequencing data analysis pipelines, meeting the requirements of researchers handling large datasets. To evaluate the GFF-Ex performance with other publicly available stand-alone parsers, we have performed comparison with functions of few parsers performing similar tasks. We compared GFF-Ex with other publicly available stand-alone gff parsers (accessed on 27-02-2013), namely, Sanger Institute GFF Perl modules [8]; Josep Abril’s GFF programs [9]; BioPerl [10]; Cufflinks 2.0.0 [11] and Galaxy module [12], to check GFF-Ex performance with its counterparts, in terms of parsing diverse features (Table 1). Most of the parsers parse sequences corresponding to only those features whose boundaries are explicitly specified within a GFF file. However, more often sequences based on other or inferred features are desirable, depending on requirements of the secondary analysis being performed. While Galaxy and GFF-Ex are able to extract sequenced based on intron boundaries, only GFF-Ex is able to define intergenic and user-defined region, upstream to gene boundaries and parse the corresponding sequences. The simple design of GFF-Ex facilitates its use by end-users with moderate or no software programming background.

**Table 1:** Comparison of GFF-Ex with other tools

	**Galaxy**	**Cufflinks**	**BioPerl**	**GFF-Ex**	**Sangers’**	**Josep Abrils’**
**Intergenic**	N	N	N	Y	N	N
**Gene**	Y	Y	Y	Y	Y	Y
**Exon**	Y	Y	Y	Y	Y	Y
**Intron**	Y	N	N	Y	N	N
**CDS**	Y	Y	Y	Y	Y	Y
**mRNA**	Y	Y	Y	Y	Y	Y
**UTRs**	Y	Y	Y	Y	Y	Y
**UpstreamToGene**	N	N	N	Y	N	N

GFF-Ex is freely available from http://bioinfo.icgeb.res.in/gff/. Additionally, GFF-Ex compatible plugins assist GFF-Ex backbone to perform customized GFF data parsing. One such example plugin developed by us is “*COLx”* (available at http://bioinfo.icgeb.res.in/gff/gffdownloads/COLx.tar.gz). “COLx” plugin exports feature based coordinates from specified columns and translates them into sequences using GFF-Ex. Another plug-in “*FETx”* (http://bioinfo.icgeb.res.in/gff/gffdownloads/FETx.tar.gz) is a plugin for feature e*x*traction, which extracts sequences on the basis of user-specified custom features declared in the input GFF annotation file. Keeping in mind the growing rates of genomic data and GFF files, the next version of GFF-Ex (under development) shall be made compatible with high performance clustered nodes.

## Conclusion

With the rapid advancements and automation of sequencing projects, the interests of scientific groups involve studies and experimental designs that make use of sequencing and GFF annotations. Handling large datasets to parse desirable information, especially sequence files, have always been an important and complex startup step in such studies. GFF-Ex is a reliable GFF parsing tool, which integrates with various applications and pipelines requiring large volumes of sequence extraction from GFF files, based on customized or GFF defined annotations.

## Availability and requirements

**Project Name:** GFF-Ex (A genome feature extraction package)

**Project home page:**http://bioinfo.icgeb.res.in/gff/

**Operating system(s):** UNIX

**Programming Language:** PERL and UNIX Shell

**License:** NA

**Any restrictions to use by non-academics:** None.

## Competing interests

The authors declare that they have no competing interests.

## Authors’ contributions

DG and AR conceptualized development of the package. DG guided AR to develop the package; AR and DG tested the package. DG and AR wrote and approve the manuscript.
